# Human Herpesvirus-6A/B Binds to Spermatozoa Acrosome and Is the Most Prevalent Herpesvirus in Semen from Sperm Donors

**DOI:** 10.1371/journal.pone.0048810

**Published:** 2012-11-07

**Authors:** Maja D. Kaspersen, Peter B. Larsen, Emil Kofod-Olsen, Jens Fedder, Jesper Bonde, Per Höllsberg

**Affiliations:** 1 Department of Biomedicine, Aarhus University, Aarhus, Denmark; 2 Cryos International Sperm Bank, Aarhus, Denmark; 3 Fertility Clinic, Odense University Hospital, Odense, Denmark; 4 Department of Pathology and Clinical Research Center, Copenhagen University Hospital Hvidovre, Hvidovre, Denmark; Ghent University, Belgium

## Abstract

An analysis of all known human herpesviruses has not previously been reported on sperm from normal donors. Using an array-based detection method, we determined the cross-sectional frequency of human herpesviruses in semen from 198 Danish sperm donors. Fifty-five of the donors had at least one ejaculate that was positive for one or more human herpesvirus. Of these 27.3% (n = 15) had a double herpesvirus infection. If corrected for the presence of multiple ejaculates from some donors, the adjusted frequency of herpesviruses in semen was 27.2% with HSV-1 in 0.4%; HSV-2 in 0.1%; EBV in 6.3%; HCMV in 2.7%; HHV-6A/B in 13.5%; HHV-7 in 4.2%, whereas none of the samples had detectable VZV or HHV-8. Subsequently, we examined longitudinally data on ejaculates from 11 herpesvirus-positive donors. Serial analyses revealed that a donor who tested positive for herpesvirus at one time point did not necessarily remain positive over time. For the most frequently found herpesvirus, HHV-6A/B, we examined its association with sperm. For HHV-6A/B PCR-positive semen samples, HHV-6A/B could be detected on the sperm by flow cytometry. Conversely, PCR-negative semen samples were negative by flow cytometry. HHV-6B was shown to associate with sperm within minutes in a concentration dependent manner. Confocal microscopy demonstrated that HHV-6B associated with the sperm head, but only to sperm with an intact acrosome. Taken together, our data suggest that HHV-6A/B could be transported to the uterus via binding to the sperm acrosome. Moreover, we find a 10 times higher frequency of HHV-7 in semen from healthy individuals than previously detected. Further research is required to determine the potential risk of using herpesvirus-positive donor semen. Longitudinally analyses of ejaculate series indicate that implementation of quarantine for a donor shown to shed a herpesvirus is not a tenable solution.

## Introduction

Semen is a known vehicle of infectious agents. Human herpesviruses have previously been detected in human semen, albeit with varying frequencies in different studies. Most studies have used sperm from men attending fertility clinics, but whether herpesviruses impair fertility is controversial and thus it remains an open question whether data from this population reflects the frequency of herpesviruses in semen from the general population. A recent study suggested that although human cytomegalovirus (HCMV) may produce a gametotoxic effect, the frequency of infectious activity was not different among semen from infertile and fertile men [1].

The initial analysis on the presence of all herpesviruses on German men seeking fertility evaluation by Bezold et al. [2], found one or more herpesviruses in 18.7% of semen samples. Epstein-Barr virus (EBV) was present in 7.1% of samples, being the most prevalent herpesvirus, followed by human herpesvirus (HHV)-6A/B in 4%, HCMV in 3.6%, HSV-1/−2 in 3.2%, and HHV-7 in 0.4% of samples. Varicella zoster virus (VZV) and HHV-8 were not detected at all. Most of the previous and subsequent studies have not been as comprehensive, but rather examined one or a few of the herpesviruses. However, these studies have found comparable frequencies in men from fertility clinics in France, UK, Massachusetts, and the Ivory Cost [3]–[9].

Notably, studies from Greece on either normal semen samples or semen samples from men attending fertility clinics report much higher frequencies, in particular of EBV (16.8–45.0%) and HCMV (7.1–62.5%) [10]–[12]. In individual studies, almost half of the samples were positive for HSV [10], and approximately two thirds were positive for HHV-6A/B [11]. Moreover, a study from Japan found HSV in almost a quarter of the samples from men attending a fertility clinic. Whether these discrepancies are caused by differences in methodology or are reflecting geographical variations remain to be determined.

Although their presence has been established, the localization of most of the herpesviruses in semen has yet to be determined. _ENREF_16 It has been reported that HSV-2 and HCMV can associate with the sperm membrane [13], whereas others have found that HSV-2 can be internalized into heads of morphological normal and motile sperm [14], [15]. HCMV may enter the germ cells at an early time point. In vitro studies with HCMV using a testis organotypic culture allowed identification of HCMV in immature germ cells by immunostaining and electron microscopy. These cells could develop into mature spermatozoa, with a concomitantly decrease in immature germ cells, implying a deadly effect of HCMV on immature male germ cells [1].

Since herpesviruses are characterized by their ability to cause latent infections that may later recur, we wished to determine the frequencies of herpesviruses in Danish donor semen, in addition to their presence in semen during longitudinal analyses.

## Materials and Methods

### Ethics Statement

The study was conducted according to the Helsinki declaration and sperm donors had signed a declaration of consent stating that the donated semen may be used for scientific intentions. Samples were anonymized and data are not referable to the donor. The Ethical Committee of Science for the Region of Middle Jutland has approved the study (M-20100238).

### Donors and Samples

Semen samples were obtained from Cryos International Sperm Bank in Aarhus, Denmark. The age of the donors at the time of sperm donation ranged from 18 to 48 years, with a mean age of 26 years. Donors had been examined and excluded if they had a predisposition to certain inheritable diseases (http://dk.cryosinternational.com/clinics/screening.aspx), previous known genital infections, current infection of *C. trachomatis* or *N. gonorrhoae* (swab), or sero-positivity towards human immunodeficiency virus type1 and 2, human T-cell lymphotropic virus type 1 and 2, hepatitis B and C, and *T. pallidum*. The ethnicity of the donors reflects that of the general Danish population of young men [16]. For storage at −196°C until further analysis, semen samples were mixed with 0.33 ml SpermCryo Allround (SCA) freeze medium (ProVitro, Odense, Denmark) per 1 ml semen.

### DNA Extraction

DNA was extracted using a Qiagen DNA extraction kit (Qiagen, Copenhagen, Denmark) and the robotic QiaCube (Qiagen) according to the manufacturer’s instructions.

### Array-based Detection of Human Herpesviruses in Semen

Herpesviruses were detected by a Clart®Entherpex kit (Genomica, Madrid, Spain) according to the manufacturer’s instruction. This kit identifies all known human herpesviruses, but does not discriminate between HHV-6A and HHV-6B. When the specific type of HHV-6 is not known, we will refer to it by HHV-6A/B. The Entherpex kit is based on amplification of a type-specific DNA fragment using primers that recognize highly conserved regions. Two different sets of biotinylated primers were used to amplify the specific fragments, which were hybridized to type-specific probes on the array. Amplified complimentary viral DNA fragments were visualized with the use of a streptavidin-peroxidase conjugate and o-Dianisidine. The platform includes an internal amplification control, which provides information of potential PCR-inhibition. Results were obtained using an Array CCD camera-reading device (Inydia, Spain), with automated interpretation and alignment software.

### Analysis of HHV-6B Binding to Sperm

HHV-6B binding to sperm was assessed by flow cytometry and confocal microscopy. Sperm samples were washed, resuspended in PBS, and purified with PureSperm (Nordic Cell, Copenhagen, Denmark) when indicated. Washed or purified sperm were resuspended in PBS to a concentration of 8 x 10^6^ per ml. HHV-6B was prepared as previously described [17] and unless otherwise noted, HHV-6B was added at 400 IU/ml (corresponding to a 1∶10 dilution of the stock), and incubated with sperm at 37°C for up to two hours. The samples were washed in PBS/2% FCS divided and stained with antibodies against HHV-6B glycoprotein (gp) 60/110 (Millipore Corporation, Temecula, CA, USA) or a control antibody against IgG_2b_ (Sigma-Aldrich, Brøndby, Denmark), followed by a secondary chromophore-conjugated goat anti-mouse (GaM) antibody (Beckman Coulter, Brea, CA, USA). The samples were then washed twice, dissolved in PBS/1% paraformaldehyde, and 50,000 sperm per sample were analyzed on a Beckman-Coulter FC500 Analyzer. From the samples prepared for flow cytometry analysis, an aliquot of 10 µl was analyzed on a Zeiss confocal microscopy with a 63× magnification. For the analysis of HHV-6B binding to sperm acrosomes, sperm were treated with dimethyl sulfoxide (DMSO) diluted 1∶100 or 10 µM calcium ionophore (in DMSO, diluted 1∶100, Sigma-Aldrich, Brøndby, Denmark) at 37°C for 30 min, then washed and incubated at 37°C with 400 IU/ml HHV-6B for 1 hr. Samples were washed, stained and incubated with 1% paraformaldehyde as described above, only this time with co-staining using rhodamine-conjugated pisum sativum (PSA) (Vector Laboratories, Burlingame, CA, USA).

## Results

### Quantification of Viruses Shed in Donor Semen

Ejaculates (n = 318) from a total number of 198 different donors were analyzed for the presence of human herpesviruses. Fifty-five of the donors had at least one ejaculate that was positive for one or more human herpesvirus. Of these 27.3% (n = 15) had a double herpesvirus infection. Since more than one ejaculate was examined for 56 of the donors, these donors had an increased probability of a positive sample. We therefore calculated the frequency by adjusting for the number of ejaculates. Thus, a donor was weighed by the fraction of positive ejaculates, ie. the number of positive ejaculates divided by the total number of ejaculates from that donor. Calculated this way, the adjusted frequency for herpesvirus in donor sperm was 27.2%.

**Figure 1:**
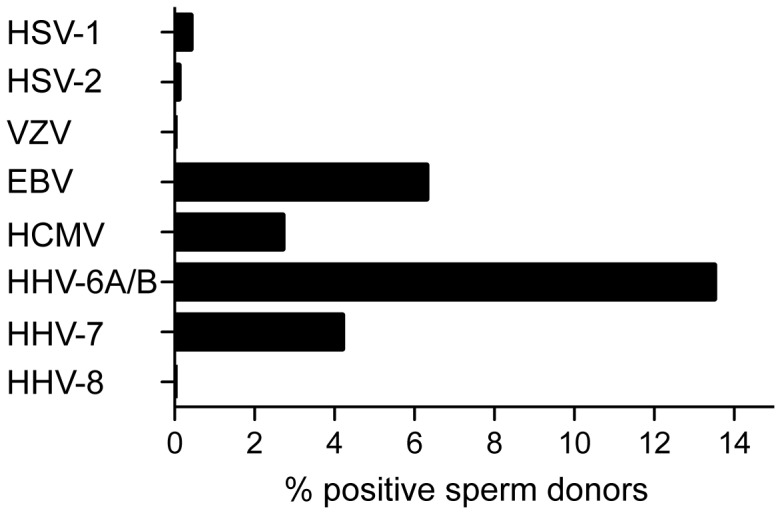
Semen samples from 198 Danish sperm donors were analyzed for presence of human herpesviruses. The viruses were detected by PCR amplification of a conserved region of the human herpesvirus genome followed by hybridization to an Array containing binding spots specific for each herpesvirus in triplicate (the Clart Entherpex kit). The frequencies were adjusted for multiple ejaculates per donor.

Similar adjustments for the individual human herpesviruses resulted in the following frequencies: HSV-1 in 0.4%; HSV-2 in 0.1%; EBV in 6.3%; HCMV in 2.7%; HHV-6A/B in 13.5%; HHV-7 in 4.2%, whereas none of the samples had detectable VZV or HHV-8 (Figure 1).

### Continuity of Viral Shedding

**Figure 2:**
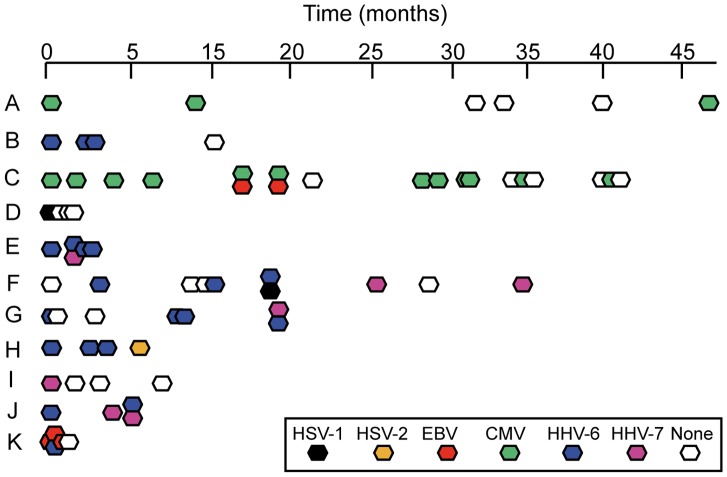
Analysis of whether shedding of herpesviruses in semen is continuous at a detectable level. Series of ejaculates from eleven different donors were screened for presence of the eight herpesviruses using PCR amplification of a conserved region of the human herpesvirus genome followed by hybridization to an Array containing binding spots specific for each herpesvirus in triplicate (the Clart Entherpex kit). Colorless hexagons represent negative samples, while virus type in a positive sample is given by different colors. Vertically contiguous hexagons indicate double infections.

In order to determine whether an ejaculate from a donor with a previously positive ejaculate can rightfully be assumed positive, series of ejaculates collected over a time period of 4 years were analyzed longitudinally from 11 herpesvirus-positive donors. This demonstrated that donors shed herpesviruses intermittently, in accordance with what is known for recurrent herpesvirus infections (Figure 2), and thus that a given ejaculate can be assumed neither positive nor negative based on previous determinations.

### Binding of HHV-6B to Sperm

**Figure 3:**
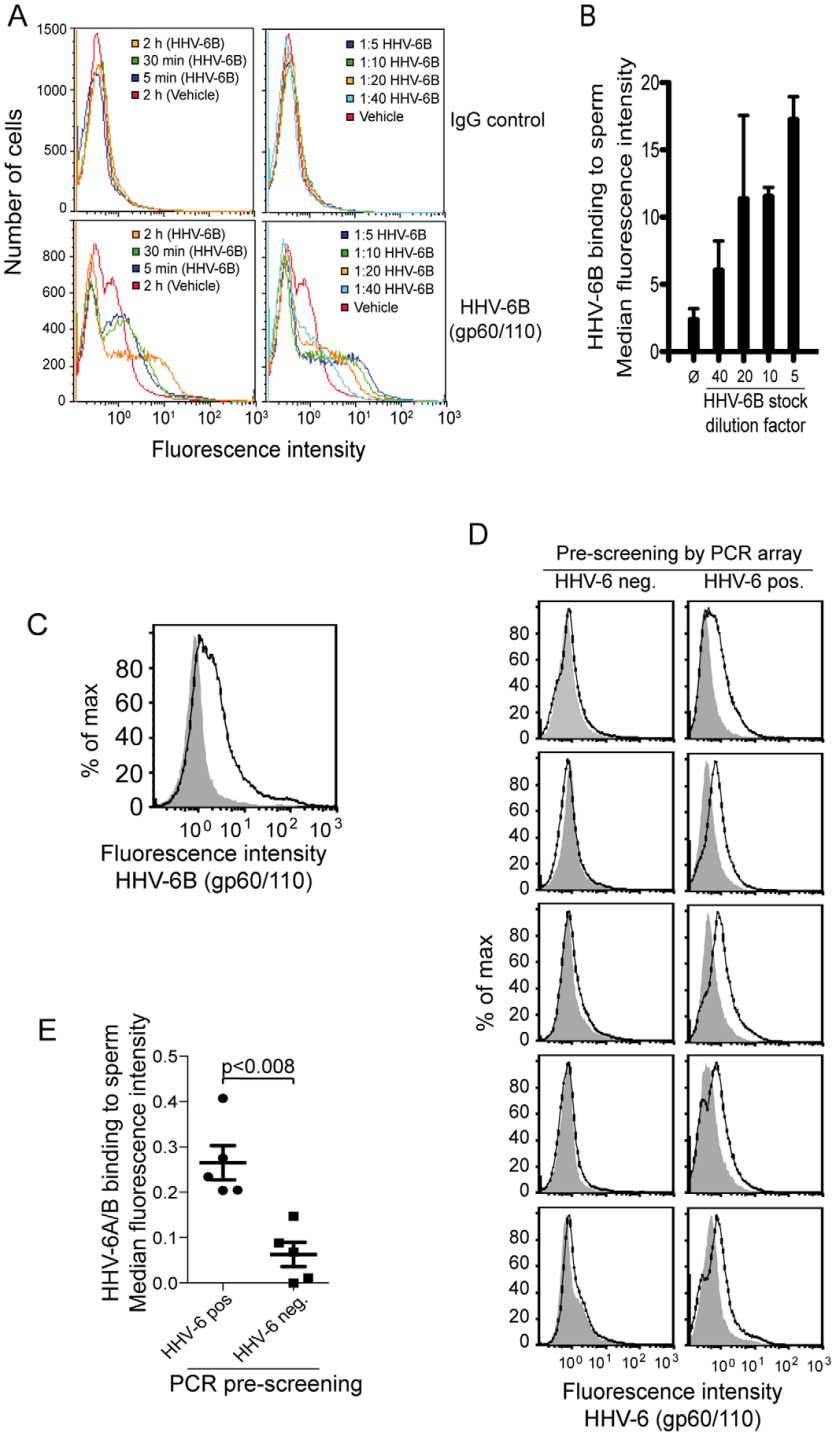
Binding of HHV-6B to sperm. Identification of HHV-6B binding to sperm was assessed by flow cytometry, analyzing 50.000 sperm per sample. (A) Thirty straw of semen were pooled and purified with PureSperm prior to incubation with HHV-6B. After incubation the sperm were washed twice in PBS/2%FCS and stained for the presence of HHV-6B with anti-gp60/110 (lower two panels), and anti-IgG_2b_ as a control for binding specificity (upper two panels). Vehicle was used as a negative control. (B) Quantitation of three experiments analyzing the concentration-dependent binding of HHV-6B to sperm. Samples were prepared as described in (A). SD is indicated on top of each bar. (C) One straw of semen was incubated with HHV-6B diluted 1∶10 and subsequently stained with anti-gp60/110 and anti-IgG_2b_ followed by a goat anti-mouse FITC-conjugated antibody. (D) Ten semen samples identified as HHV6A/B negative (five) or HHV-6 positive (five) by the PCR array were stained with anti-gp60/110 and anti-IgG_2b_ and analyzed by flow cytometry. (E) Plot of delta median fluorescence intensity of data shown in (D); p<0.008, Mann-Whitney test.

The most prevalent herpesvirus in semen was HHV-6A/B, of which HHV-6B is the variant known to be shed in secretions [18]. To further explore whether HHV-6B was capable of binding directly to sperm, HHV-6B was mixed with sperm pooled from straws of several donors. Potential binding was assessed by flow cytometry using an antibody to the HHV-6A/B gp60/110 (Figure 3). Incubation of sperm that were pooled from 30 ejaculates from different donors demonstrated that incubation for as little as 5 min resulted in a clearly detectable association between HHV-6B and sperm. This association was increased with incubation periods up to 2 hrs, which was the longest incubation period tested (Figure 3A, left lower panel). The association was also dependent on the amount of virus and decreased proportionally to dilution of the virus titer (Figure 3A, right lower panel and Figure 3B).

Notably, even in vehicle-treated semen samples, a low intensity binding of gp60/110 was detectable. This was not caused by unspecific antibody binding to sperm, since a single straw of sperm that tested negative for HHV-6B by PCR showed only anti-gp60/110 antibody binding if HHV-6B was added in vitro (Figure 3C). We therefore tested all the donors from the HHV-6 array where additional straws were available for the same date. This included five HHV-6A/B-negative and five HHV-6A/B-positive donors. Flow cytometry demonstrated binding of HHV-6A/B anti-gp60/110 antibody to sperm of all tested samples, in which HHV-6A/B could be detected by the HHV-6A/B PCR array, but in none of the HHV-6A/B-negative samples (p<0.008). This strongly suggests that HHV-6A/B binds to sperm *in vivo*.

### HHV-6B Binds to the Sperm Acrosome

**Figure 4:**
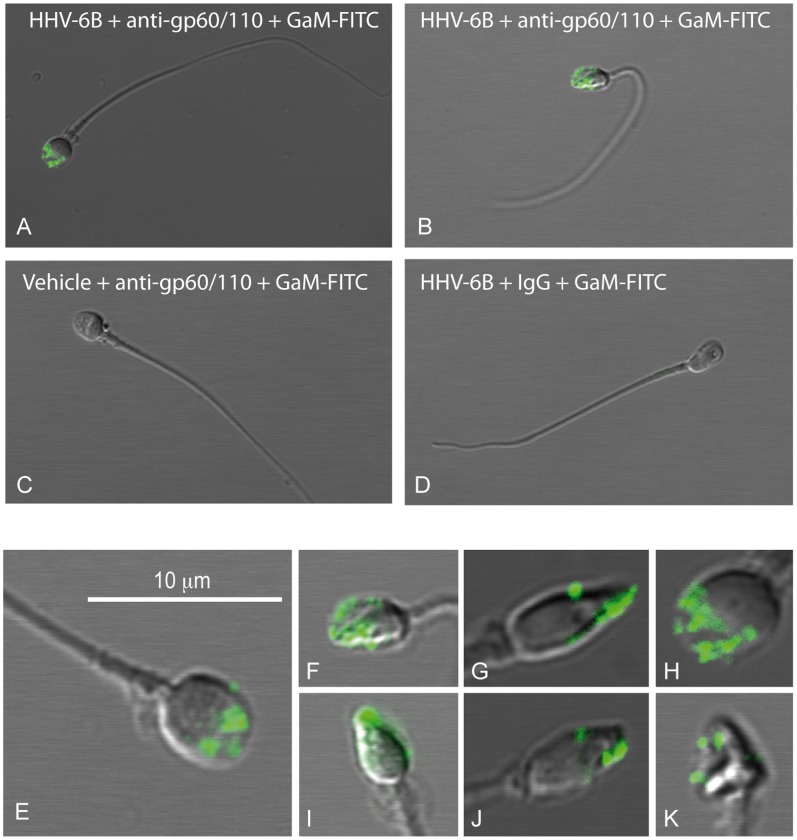
HHV-6B binds to the sperm head. Semen samples were incubated with HHV-6B for two hrs, washed twice in PBS and stained with anti-gp60/110 or anti-IgG_2b_ followed by a goat-anti mouse (GaM)-FITC. (A-B) and (E-K) HHV-6B binds specifically to the front region of the sperm head. (C) Control staining for non-specific binding of anti-gp60/110. Sperm were pre-incubated in vehicle and stained with anti-gp60/110 and GaM-FITC. (D) Control staining for non-specific binding of GaM-FITC. Sperm were pre-incubated with HHV-6B and stained with IgG_2b_ and GaM-FITC.

**Figure 5:**
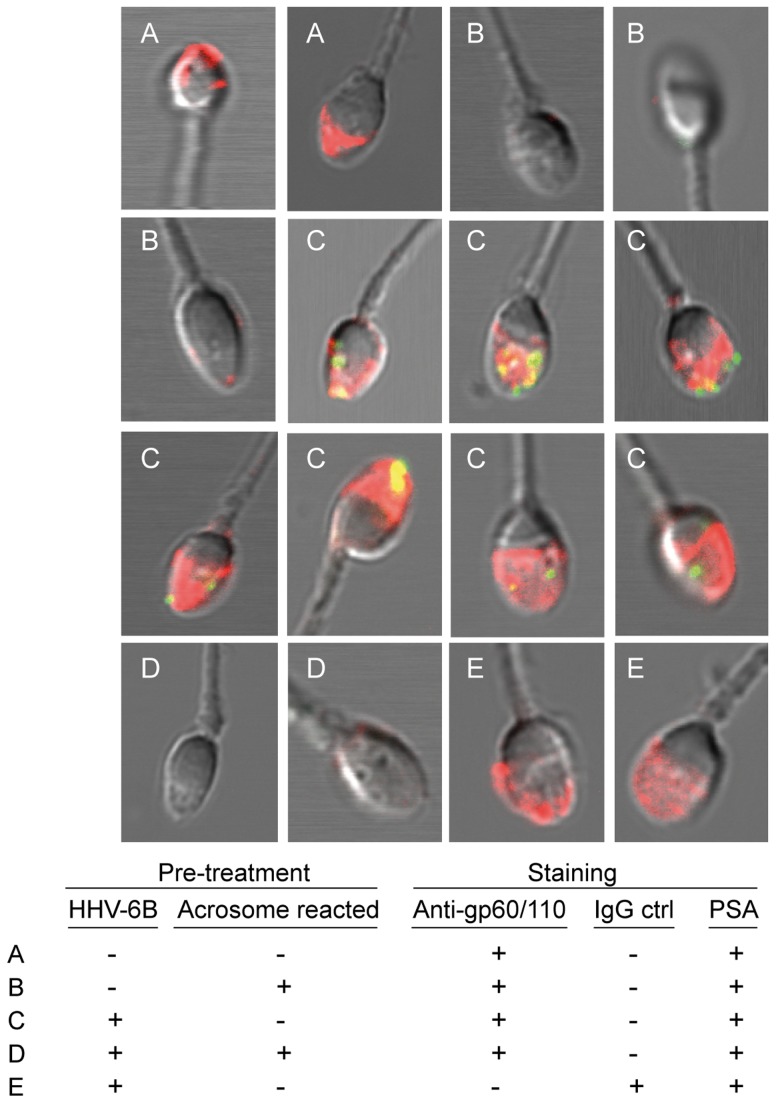
HHV-6B binds the sperm acrosome. Semen samples were pre-incubated with HHV-6B for one hr followed by incubation with either vehicle or calcium ionophore for 30 min to induce an acrosome reaction (AR). After incubation with both calcium ionophore and virus the samples were washed twice in PBS and stained with anti-gp60/110 or anti-IgG_2b_ followed by a goat-anti mouse (GaM)-FITC concomitantly with rhodamine-conjugated pisum sativum (PSA). The PSA (red) stains intact acrosomes and leaves acrosome reacted sperm unstained. (A-E) Different combinations of pre-treatment and subsequent staining are indicated at the bottom of the figure.

The detection of HHV-6B association to sperm by flow cytometry, prompted us to examine whether the virus bound to specific regions of the sperm. Sperm were incubated with HHV-6B and prepared for confocal microscopy (Figure 4). Specific binding of HHV-6B to sperm was observed within the area of the sperm acrosome (Figure 4A and B, and E-K). To further verify the dependency of the acrosome for HHV-6B binding, an acrosome reaction was induced with calcium ionophore. Staining for the presence of the acrosome using PSA demonstrated that HHV-6B only bound sperm with an intact acrosome (Figure 5). This suggests that the acrosome contains a receptor for HHV-6B, although the identity of the receptor remains to be determined.

## Discussion

The potential serious consequences of congenital infections highlights the importance of decreasing transmission of infectious agents to the mother, the fetus or the newborn. Sperm is a possible transmission route, in particular for the mother. Herpesviruses are one family of viruses with several members of importance for sexually transmitted infections. HCMV is well known for its ability to cause serious congenital infections. Although not complied with by the Danish sperm banks, guidelines for gamete donations presented by the American Society for reproductive Medicine (ASRM) in 1998 included screening of donors for HCMV IgG and IgM [19]. However, serologic screening may not be sufficient for detecting HCMV infection, and conversely, HCMV sero-positivity does not predict viral shedding in semen [8].

Additional research is needed to address the issue of whether screening of donor ejaculates for human herpesviruses should be required of sperm banks before the ejaculate is released. Our analysis of ejaculate series strongly implies that implementation of quarantine for a donor shown to shed a virus is not a tenable solution. Donors with a sperm sample positive for a herpesvirus may later become non-detectable for the same virus, which again may recur in a later sample. This suggests that each sperm sample rather than the donor should be characterized for the presence of virus.

We find that HHV-6A/B is the most prevalent herpesvirus among donors from Denmark. HHV-6A/B is a beta-herpesvirus and thus related to HCMV. Whether HHV-6A/B may cause some of the same problems for pregnant women as is known for HCMV is poorly understood. We found that HHV-6B is able to bind to the acrosome of the sperm, but it is still an unsolved question how herpesviruses come into contact with the mature sperm. This might occur in the testis during spermatogenesis or from an infection of the urogenital tract. Although Naumenko et al. [1] have provided evidence that HCMV is capable of infecting immature germ cells, our demonstration of HHV-6B binding to sperm within minutes suggest that the latter option is also possible. There is no evidence of HHV-6A/B infection of testes and thus it may be more likely that sperm are associated with HHV-6A/B during ejaculation. In contrast to HHV-6A, HHV-6B is known to be frequently present in secretions. Thus, we believe that the detected HHV-6A/B by the PCR array is indeed HHV-6B. PCR tests on selected donor samples with HHV-6B-specific primers confirmed the presence of HHV-6B in semen (data not shown).

The consequences of herpesviruses on sperm quality or human reproduction, if any, are still debatable. However as long as we are not certain that these viruses are harmless, we must acknowledge the risk of viral transmission through semen. In particular, whether HHV-6B may be transmitted to the oocyte by means of the sperm requires further analysis. Our finding that HHV-6B binds to the acrosome may argue against this possibility, since the acrosome is dissolved when the sperm interacts with the egg. Nevertheless, HHV-6A/B has been reported to be chromosomally integrated [20] at a frequency as high as 0.8% of the UK population [21], and it remains unknown how this integration might happen. The binding to the acrosome also suggests the possibility that HHV-6B may be transmitted to the uterus by the sperm. Whether HHV-6B is able to infect cells in the uterus should also be investigated further. Indeed, it has been suggested that HHV-6A/B infection may predispose to spontaneous abortion and HHV-6A/B was found in abortive villous tissue [22]. The recurrent nature of herpesvirus infections may require longitudinal analyses to address whether HHV-6A/B infects and is secreted from cells within the uterus.

We used a sensitive PCR test to determine the frequency. However, our longitudinal analysis indicated that donors might change their status of herpesvirus secretion with time. Since virtually all adults are HHV-6A/B seropositive, it is possible that almost everybody may secrete HHV-6A/B in semen at some point, although the level may vary considerably between individuals, which may be important. Clearly, it becomes a challenge to determine the potential consequences, if any, of the intermittent shedding of a common virus.

Importantly, the binding of HHV-6B appeared to be specific since it was only seen on sperm with acrosomes. Despite using a high titer of HHV-6B, Figure 3A reveals a population of sperm not susceptible to HHV-6B binding. This very likely represents acrosome-reacted sperm. The fraction of sperm in an HHV-6A/B-positive sample that binds anti-gp60/110 varies between samples (Figure 3C–D), as does the fraction of spontaneously acrosome-reacted sperm.

HHV-6A/B was detected by flow cytometry on sperm that were also HHV-6A/B positive by PCR. Moreover, approximately one out of seven men secretes at a given point HHV-6A/B in semen. Thus, it is not surprising that the vehicle-treated sample in Figure 3A, which is based on a pool of 30 different semen straw from different donors, shows binding of anti-gp60/110.

Besides HHV-6A/B, our study finds the related virus HHV-7 in approximately 1 out of 24 semen samples from healthy donors. To our knowledge, HHV-7 has not previously been detected in semen from normal donors and thus its significance, if any, is unknown. Bezold et al. found that 0.4% of German men seeking fertility evaluation had HHV-7 in their semen [2], but Neofytou did not detect HHV-7 in 172 individuals, 92 of who had abnormal semen samples [11]. A potential role of infection with HHV-7 is via influence on other herpesviruses, e.g. by transactivation of genes from the related HHV-6A/B or HCMV.

In summary, our study has identified several herpesviruses, of which HHV-6A/B was the most predominant, in semen from healthy donors. The virus appears to be transmitted bound to the acrosome of sperm. Moreover, we demonstrate that the presence of herpesviruses may fluctuate, even for the same donor, probably reflecting recurrence of the latent infection.
